# Pre- and post-COVID-19: The impact of the pandemic and stock market psychology on the growth and sustainability of consumer goods industries

**DOI:** 10.3389/fpsyg.2022.796287

**Published:** 2022-11-18

**Authors:** Naveed Jan, Zeyun Li, Liu Xiyu, Muhammad Farhan Basheer, Korakod Tongkachok

**Affiliations:** ^1^Department of Management science and Engineering, Business School, Shandong Normal University, Jinan, China; ^2^School of Humanity, Universiti Sains Malaysia, George Town, Malaysia; ^3^Lahore Business School, University of Lahore, Lahore, Pakistan; ^4^Department of Law, School of Law, Thaksin University, Songkhla, Thailand

**Keywords:** pre and post-COVID-19 pandemic, psychology of stock market, business units, trade volume, market reaction

## Abstract

The objective of this study is to investigate the impact of the COVID-19 pandemic and stock market psychology on investor investment decisions in different business units operating in the Shandong stock market. The sample size of the study consists of 5,000 individuals from six different business units. The study used the event study statistical technique to analyze the market reaction to newly released information from the stock market perspective to assess whether the number of COVID-19 positive cases impacted it. With a *Z* score value of 40.345 and a *P*-value of 0.000, the Wilcoxon test indicated that stock prices before and after the pandemic were quite different. The test showed a positive relationship between the pandemic and the stock market. Further, the results indicated that COVID-19 and stock market psychology had a significant positive impact on investor investment decisions in cosmetic and beauty, consumer household, textiles and apparel, and consumer electronics industries; however, in the sporting and consumer appliance industries, it had an insignificant negative impact. This study serves to guide investors to make suitable changes in their stock market trading practices to counter these challenges to increase their required rate of return from their specific stock market investment. The findings have important insights for various stakeholders including governments, regulatory bodies, practitioners, academia, industry, and researchers.

## Introduction

Toward the end of 2019, the coronavirus disease (COVID-19) caused by a novel SARS-CoV-2 virus began threatening the physical health and lives of millions of people around the world, resulting in large-scale deaths within a few weeks. The highly contagious and rapidly transmitting nature of the coronavirus caused widespread anxiety, worry, and concern in the general public resulting in an overall decline in psychological health worldwide [World Health Organization (WHO), 2020]. As a result, the WHO developed strategies for dealing with the condition from both a medical and psychological standpoint. Various studies on COVID-19 have focused on virus propagation, containment methods, and potential vaccines, and researchers are just beginning to explore its impact on mental health and possible treatment. Increased anxiety appears to be the most prevalent symptom followed by sadness and sleep difficulties according to a new study on the Chinese population (Huang and Zhao, 2020). In early 2020, large numbers of Chinese organizations and research groups began looking into the pandemic's overall negative psychological impact.

In a detailed online survey conducted at the start of the outbreak in China it was revealed that nearly half of the respondents were suffering from moderate to severe psychological effects; 29% of respondents reported moderate to severe anxiety symptoms, 17% reported moderate to severe depressive symptoms, and 9% reported moderate to severe stress levels (Wang C. et al., 2020). Another cross-sectional study found that a month after the pandemic the incidence of post-traumatic stress symptoms in China's Hubei region was close to 10% with higher rates in women and those with poor sleep quality (Liu et al., 2020). A third study used machine-learning predictive models to analyze posts from Weibo (a popular Chinese social media platform) and found that after the announcement of COVID-19 on 20 January 2020, negative emotions (e.g., anxiety, depression, indignation) and sensitivity to social risks increased while positive emotions and life satisfaction decreased (Li et al., 2020). The pandemic has spread to over 200 countries impacting both human wellbeing and commercial sectors of the global economy. According to Dow Jones and Standard and Poor's, corporate stock prices in the United States declined 20% in mid-March 2020. Similarly, the stock prices of companies dropped dramatically on Nikkei. Sri Lanka's stock market exchange saw a 10% drop in its index stock prices forcing it to close transactions multiple times in March 2020. Though China was able to contain the COVID-19 outbreak, its productivity and economy were severely hit. During the pandemic-triggered lockdown, operations in businesses and factories in China were essentially scaled down but not completely shut down. As a result, in the first 2 months of 2020, the country's manufacturing profit fell by 38.3%. During the same period, 5 million people lost their jobs with urban unemployment rising to 6.2% in February 2020 up from 5.2% in December 2019. Several psychological factors influenced consumer behavior according to previous studies in consumer psychology and behavioral economics (Durante and Laran, 2016). Consumer behavior is the study of individuals or groups who are looking for products and services to meet their requirements and are looking to buy, use, assess, and dispose of them (Rajagopal, 2020). It is also important to look at the consumer's emotional, mental, and behavioral responses that occur before or after these procedures (Kardes et al., 2011). Changes in consumer behavior can be caused by a variety of factors, including personal, economic, psychological, cultural, and societal aspects. However, in severe situations such as a disease outbreak or a natural disaster some elements have a greater impact on consumer behavior than others. Situations that threaten people's health or disturb their social lives have been shown to cause significant behavioral changes (Leach, 1994). Panic buying is a phenomenon that occurs when fear and panic impact behavior, causing consumers to buy more than usual (Lins and Aquino, 2020). Panic buying has been defined as a herd behavior in which consumers purchase a large number of things in advance during or after a calamity (Steven et al., 2014). Similar changes in consumer behavior occur when purchasing decisions are clouded by negative emotions such as fear and anxiety (Yuen et al., 2020). Lins and Aquino (2020) found that panic buying was positively correlated with impulse buying in the context of the COVID-19 pandemic. In this instance, it has been defined as a complex buying behavior in which the rapidity of the decision process prevents thoughtful and deliberate consideration of alternative information and choice (Yuen et al., 2020). To better understand consumer behavior in stressful conditions a recent study suggested distinguishing between necessity and non-necessity products (Durante and Laran, 2016). According to previous studies, the disparate findings on the link between stress and consumer behavior could be attributed to the fact that stress has a detrimental impact on purchasing habits of some while having a beneficial impact on others depending on the type of product being considered. On the one hand, it has been suggested that by making everyday survival supplies readily available customers will be more ready to spend money on essentials (rather than non-necessities). As a result, a recent study has found that purchasing essentials (i.e., utilitarian shopping) increases during and after a traumatic occurrence (Larson and Shin, 2018). However, other findings reveal that impulsive nonessential purchases (i.e., hedonic shopping) may rise as a means of escaping or minimizing the suffering associated with the scenario; i.e., purchasing non-essentials is used as an emotional coping mechanism to deal with stress and unpleasant emotions (Kemp et al., 2014). Durante and Laran (2016) hypothesized that consumers engage in strategic consumer behavior to regain control in stressful times to reconcile their sense of lack of control. As a result, consumers with high stress levels are more likely to conserve money and spend strategically on things they perceive as necessities. Importantly, when it comes to the impact of perceived stress from the COVID-19 pandemic on consumer behavior a recent study found that higher levels of perceived stress increased the likelihood of purchasing bigger quantities of food (Jezewska-Zychowicz et al., 2020). Rehmani et al. (2021) conducted a study to find empirical evidence on whether work from home or residential emissions reduces office emissions, and found that emissions stayed in the environment of employees' workplace in both online and offline conditions. Employees were least affected by the various emissions as they worked online instead of working at organizational setups.

The current study explores how the stock price and volume of trading changed causing a significant shift in consumer habits, intentions, and frequency of purchase and their subsequent impacts on investors' investment decisions concerning different consumer goods industries'.

It has several contributions, including theoretical contributions for various shareholders. The study brings fresh perspectives to the limited literature by providing new insights about pre and post-COVID-19 and stock market psychology impacts on the stock markets and investor investment decisions in the consumer goods industry. The study also contributes by determining Chinese investors' pre and post-COVID-19 stock market psychology on investment frequency because China was the first country to recover from the global pandemic.

In March 2020, the International Monetary Fund projected that “C'ina's slowdown in the first quarter of 2020 will be substantial and will leave a deep mark for the year” as the Chinese economy sunk to an all-time low in the first 2 months of the year (Hoehl et al., 2020). In reality, the Chinese economy dropped 6.8% in the first quarter of 2020 compared to the previous year (Liew and Puah, 2020). From the perspective of the stock exchange and indicator of economic growth, the news of the COVID-19 outbreak led Chinese stock markets to fall significantly (Analytica, 2020). The Shanghai and Shenzhen composite indices suffered to a large extent in single-day losses (2.75 and 3.25%, respectively). On 3 February 2020, these two indices experienced another 8% fall in the opening session. Both composite indices ended the day with losses of 7.72 and 8.45% respectively, which were the largest single-day losses in the past 12 months (Han et al., 2020). The current empirical study aims to provide a detailed measure of the impact of the COVID-19 outbreak on the Chinese stock market. Market efficiency is divided into three basic types: - weak-form efficient market, mid-high efficient market, and high efficient market (Nilsson and Bylund Månsson, 2019). The market is considered to be in deprived shape when market information and price are completely expressed by the securities price rather than future price. When all publicly accessible information is completely expressed in security price the market is considered a mid-strong efficient market. When all information, both individual and set apart, is revealed in the security market the market is deliberated as an efficient market. The efficient market hypothesis is performed to determine weak and mid-strong markets. When investors are unable to obtain abnormal returns using all available and historic information in the stock market, that results in a semi-strong efficient market hypothesis. The effectiveness of a semi-strong market is assessed by retributions announcement data and the company's stock price's rapid change. The Chinese market could be in the middle of a peak when information about an occurrence is recognized by the shareholders. This episode is analyzed to assess reactions and market response. The reaction can be evaluated by the stock's closing price. A shift in security trading size reflects stakeholder decisions. Evidence is essential for stakeholders and industry actors as it allows them to describe the current and potential state of the market. Investors and market players may benefit from the completeness, precision, and timeliness of knowledge while making investment decisions. Information sent out in such situations can be positive and negative.

### The impact of the pandemic and stock market psychology on capital market

The COVID-19 pandemic impacted capital market practices in all countries around the world. Investors generally use the information embedded in such events to make investment decisions. The shift in stock price and stock exchange volume indicate the capital market's reaction. Ramelli and Wagner (2020) studied how the marketplace was forced to react to the'pandemic and the commercial implications it had. Several kinds of research on consumer reactions to pandemics have been carried out (Donthu and Gustafsson, 2020). Fallon and Sarmiento (2021) explored the relationship between pandemic cases and stock market trends. Machmuddah et al. (2020) predicted a 50% security value drop during the pandemic, but a fast recovery thereafter once the short-term shock of labor supply in the market eased. The stock price exhibited a W-shaped pattern under the optimal strategy and remained around 10% underrated for the better part of a year. Ahundjanov et al. (2020) predicted that a global pandemic death rate of more than 1% will result in a 0.02% decline in the SP500 after 1 day by 10% after 1 week and 10% after a month in the S&P 500. Alfaro et al. (2020) studied typical infectious disease models that lead to day-to-day unexpected variations in expected circumstances. Their results indicated that stock market unpredictability would decrease as the pandemic's trend becomes less uncertain. Stock prices responded more rapidly and intensely in nations affected by the 2003 severe acute respiratory syndrome (SARS) outbreak although stock price responses were resilient in nations with a greater debt-to-GDP ratio (Hanke et al., 2020). Ramelli and Wagner (2020) showed that the COVID-19 health index was generally favorable from the perspective of the stock market investment. They looked at the effect of social distancing policies on economic activity and stock market indices. Their findings showed that the increasing number of lockdown days, monetary policy decisions, restrictions on international travel, and the closing and opening of the lowest and highest stock prices of major stock market indices, all had a significant effect on economic activity. Internal movement restrictions had a positive effect on economic growth, as did increased fiscal policy expenditure. On the other hand, the increasing number of coronavirus cases had no discernible effect on economic activity (Hasan et al., 2021). The findings of Cookson and Niessner (2020) were found to be incorrect in several commercial markets. Due to the reduced severity of COVID-19 in China, the financial markets remained strong and stable. In comparison with international markets, the industry remained relatively stable despite the pandemic's spread. This result is in line with Jiang et al. (2021) who asserted that the movements of the financial market index, especially the stock market, demonstrate how investor perception affects a specific industry. Sansa (2020) and Wang and Li (2020) observed that in China the financial markets remained strong and stable despite this severe pandemic situation. Other studies on the effect of the pandemic on price exchange trades have obtained different results. Therefore, in our study, we set out to offer a more thorough description of the severe situation and its impacts.

Hoarding is described as the act of accumulating and securing a higher number of products than is required for the future (Chu, 2018). The consequence of over-acquiring products during a crisis is a challenge for supply chain disruption risk management from an economic and social standpoint. In the past, it has been widely established that people exhibit higher hoarding behavior during times of distress. Hori and Iwamoto revealed how in the aftermath of the Tohoku earthquake in 2011, consumers adjusted their purchase behavior in favor of hoarding by identifying certain hoarder profiles (Hori and Iwamoto, 2014). Similarly, it has been proven that throughout every typhoon season in Taiwan, seasonal items are systematically hoarded (Zanna and Rempel, 2008). Moreover, during this season attitudes toward risk-driven hoarding and disaster-induced affective responses are important elements that affect the agricultural-food supply chain (Sheu and Kuo, 2020). Similarly, since the outbreak of COVID-19, people have stockpiled certain products (e.g., toilet paper, sanitizing gel) all over the world whether or not they're directly related to the pandemic (Kirk and Rifkin, 2020). Few studies in non-western societies have looked at the link between hoarding behavior and personality factors (Nowak et al., 2020). It is crucial to see if the relationships determined in studies conducted in western countries apply equally to different ethnic and cultural situations (Henrich et al., 2010). Long and Khoi (2020) studied food hoarding intentions in Vietnam but they omitted personality factors. During the COVID-19 pandemic in Japan, there was a shortage of various products. This is juxtaposed to collectivism, to which Japanese culture belongs to, where people tend to obey rules and behave in agreement with others as was evidenced by many Japanese individuals stressing social norms during the pandemic (Nakayachi et al., 2020). Perhaps then, hoarding behavior could be explained by other cultural aspects not just collectivism (De Mooij and Hofstede, 2011). For example, similar behavior may be evident in a culture of indulgence (e.g., Brazil). Additionally, other studies have indicated that when natural calamities such as earthquakes and typhoons occur, people in Japan stockpile important items (Watanabe et al., 2014). This implies that such actions are due to individual variances. As a result, personality factors may have precipitated hoarding behavior among Japanese during the COVID-19 pandemic.

The objective of this study is to determine the difference in stock market price and volume of trading before and after the pandemic and the subsequent impacts on investors' investment decisions.

This study, therefore, contributes to answering a central question: is there any difference in stock market price and volume of the trade before and after the outbreak of COVID-19?

Given the lack of comparative analysis of before and after COVID-19 stock price and stock market psychology on various business units operating in the Shandong province, this study is timely in filling this research gap. These business units are an important source of revenue generation for the province, and this study addresses the absence of research in this area. Our study indicates that substantial variations exist in the pre and post-COVID-19 stock market price and volume of trading and their subsequent impacts on investor stock market investment decisions. Overall, our results revealed that an increase in the COVID-19 positive cases had a substantial negative impact on investors' stock market investment decisions. However, a decrease in the positive cases during the pandemic period had a substantial positive impact on investors' stock market investment decisions. The results of the Wilcoxon test suggest a *Z* score value of 40.345 and *P*-value of 0.000. This suggests that stock prices before and after the pandemic were quite different. As a result, there was a positive relationship between the pandemic and stock price. Further, our results indicated that COVID-19 and stock market psychology had a significant positive impact on investor investment decisions in cosmetic and beauty, consumer household, textiles and apparel, and consumer electronics industries. However, an insignificant negative relationship was found between COVID-19 and stock market psychology and investor investment decisions in the sporting goods and consumer appliance industries.

The findings from the present study will provide valuable insights to practitioners in various dimensions of financial markets. This study is a novel approach and addition to the literature on stock price and volume of trading and their subsequent impacts on investor investment decisions before and after COVID-19 in the consumer goods industries in the context of China.

A brief review of various kinds of literature is presented in the next section, which is followed by the development of the research hypothesis. Next, we describe the specific methods used in the current study. We then describe the analysis and results followed by a detailed explanation of the research findings. The last section highlights certain limitations of the current study and provides several recommendations for future analysis.

## Literature review

### Theoretical framework and hypothesis development

The efficient market hypothesis (EMH) posits that security prices represent rapidly and accurately all available information. This specific information includes commercial, administrative, societal, and trade activities. The efficient market hypothesis reflects how information influences security prices and how the market reacts. According to Titan (2015), an efficient market responds rapidly to influence a new stock price that completely represents all available information. Based on this information there are three levels of efficient market hypothesis: (1) a weak form of efficient market hypothesis states that stock price fully and fairly reflects all historical information, (2) a semi-strong form of efficient market hypothesis states that current stock prices adjust rapidly to the release of all new public information, and (3) a strong form of efficient market hypothesis states that current stock prices reflect all new public and private information.

### Relationship between stock price event and stock market psychology

The EMH states that a market can react rapidly and accurately to new information. This information is chosen carefully by investors before making an effective investment decision in stock markets. At the time of making an effective capital market investment decision, investors must analyze the prevalent information in stock markets. According to Wang J. et al. (2020), information will not always be of the best quality. The quality of the information is influenced by the embedded content, based on which information knowledge may be classified as applicable or non-applicable to the stock market. A piece of information is useless if it is not communicated to investors on time. Lyócsa et al. (2020) investigated the positive relationship between market trends and macroeconomic and administrative information development. According to Liu and Pan (2020), the stock market and macroeconomic information have a negative relationship. Security price measure is primarily determined by innovative evidence contribution and treated in combination with the market scenario (Andersen et al., 2020). Investor confidence is unaffected by announcements that do not provide new information about the market situation (Bonsall IV et al., 2020). This finding is consistent with Galdi (2021) who demonstrated that wherever price expresses knowledge level variation, it increases the volume of trade.

### Hypothesis development

During investment decisions, investors use the most relevant information that they possess regarding the stock market investment decision. The status of the economy has an impact on the information in the stock market (Syifaudin et al., 2020). Although it is not particularly tied to capital market dynamics, the non-economic atmosphere cannot be isolated from stock market behaviors. Several factors influence the stock market's stock price. According to Khan et al. (2019), investor commitment is an influence that activates investor sentiments during decisions. Positive emotions are critical for helping investors make smarter decisions (Srivastava et al., 2021). Fashion engagement is believed to boost trade intent as well as happy sensations. Hedonic trading value is another key factor that affects involuntary decisions. A previous study linked impulse purchasing to hedonism which drives investors' desire to purchase securities (Cuong et al., 2020). Auctions marketing is the third component that leads to positive sentiments and spontaneous decisions. It is a collection of several advertising tactics that enable individuals to purchase a variety of securities immediately. According to Mohan et al. (2013), retailers who want to increase impulse buying use sales promotional activities that focus investors' attention on emotionally appealing securities. Kesari et al. (2021) stated the need to satisfy investor needs by creating positive emotions around specific investment decisions. As a result, the investor is drawn to make impulsive decisions rapidly. Investors are encouraged to put away non-scheduled securities by fluctuating positive emotions. Mental activity has a positive impact on happiness, excitement, and joy. In both domestic and foreign markets, a trader from a developing country can use social commerce platforms to reach a larger number of potential businesses; however conventional channels operate in many circumstances and are less expensive (Guan et al., 2020). When public trade is to be exploited for long-term growth, such policies must be reviewed. The policy on social commerce should be consistent and its goals should be in line with the goals of the national development plan (Molinillo et al., 2021). Similar research has presented a range of analytical methodologies for determining the fundamental elements of social media-related information. Emotional and impulsive investors are more prone to decisions on the spur of the moment (Axfors et al., 2021). Since the mid-twentieth century, the rapid growth of information technology has profoundly affected the settings both within and outside of enterprises. Companies have been embracing new ICTs as a strategic tool to boost productivity in areas including organizational goals, innovation, communication, and environmental change predictions (Acciarini et al., 2021). Consumers' IB is one of their most recognizable traits and emotions play a critical part in purchasing decisions (Chu, 2018). The COVID-19 pandemic has changed customer behavior, making anxiety a major marketing challenge. It resulted in enormous psychological, social, and economic upheavals including job losses, low earnings, and social anxiety.

**Figure 1:**
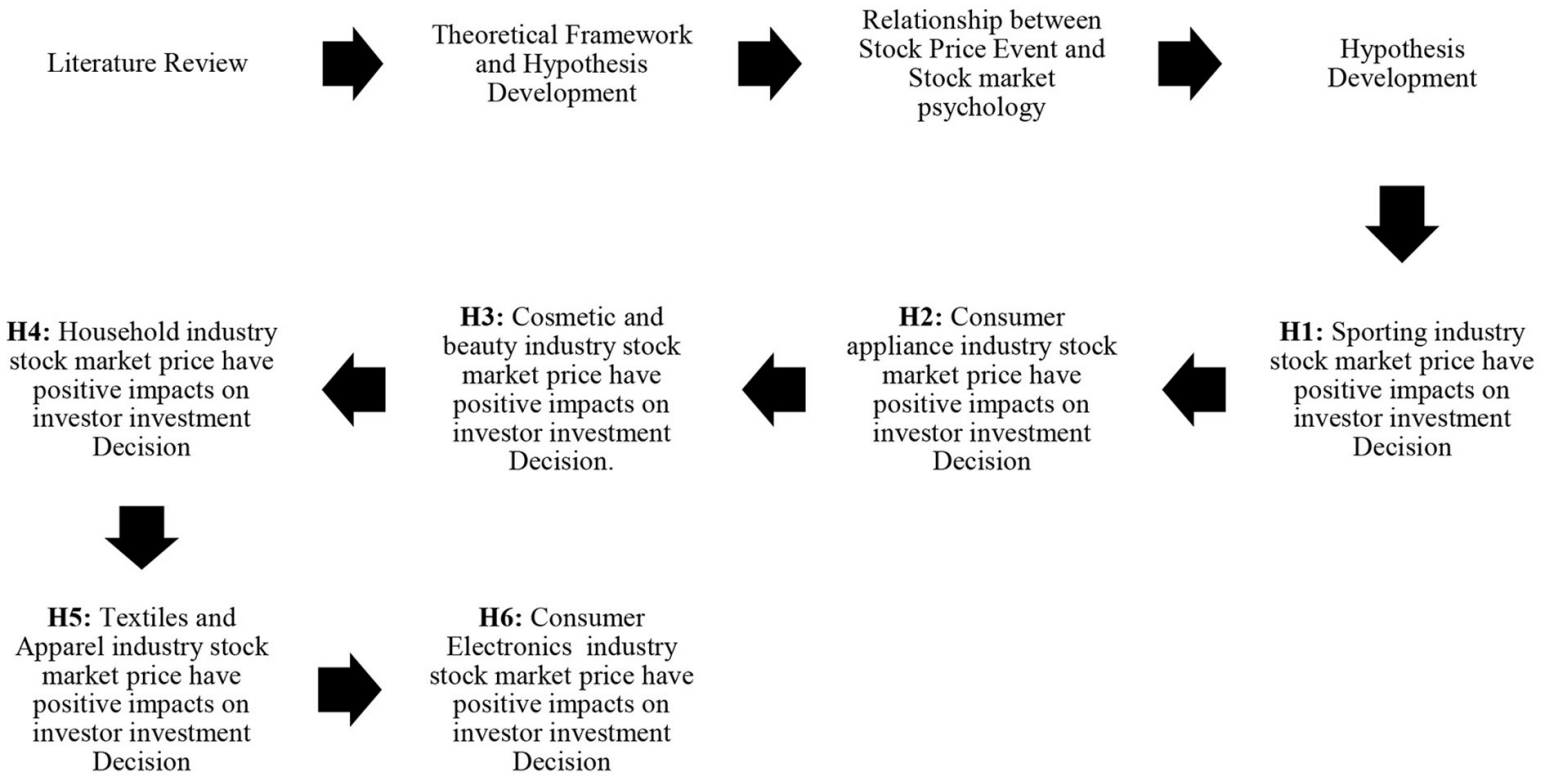
Graphical representation of literature review. Source: Author.

The COVID-19 pandemic, social isolation, and lockdown directives disrupted customers' buying, shopping, and consumption habits (Badr et al., 2020). As a result of which, people shifted to online shopping, raising the risk of impulse purchases. The pandemic was a one-time event; however, the propensity of COVID-19 to strike at any time declined the stock prices again in the stock market. The pandemic had an impact on human wellbeing and all industries worldwide. The EMH is a mathematical model that depicts how stock prices react to new information. According to the EMH, the market reacts quickly to newly released information. The evidence includes not only commercial evidence but also politically aware, societal, and fiscal activities as well as other information. In the study by Sansa (2020), the influence of the pandemic on commercial markets in two developed nations of the current world such as China and the United States was explored. The results confirmed a significant positive relationship between the number of confirmed COVID-19 cases and financial markets. Our findings are also consistent with (Machmuddah et al., 2020). According to Ramelli and Wagner (2020) the pandemic had a significant positive impact on stock market prices. Based on the above discussions the following hypotheses were formulated:

**H1:** Pre- and post-COVID-19 and stock market psychology have a significant positive impact on investor investment decisions in the sporting goods industry.
**H2:** Pre- and post-COVID-19 and stock market psychology have an insignificant negative impact on investor investment decisions in the consumer appliance goods industry. The graphical presentations is presented in above Figure 1.
**H3:** Pre- and post-COVID-19 and stock market psychology have a significant positive impact on investor investment decisions in the cosmetic and beauty goods industry.
**H4:** Pre- and post-COVID-19 and stock market psychology have an insignificant negative impact on investor investment decisions in the household goods industry.
**H5:** Pre- and post-COVID-19 and stock market psychology have a significant positive impact on investor investment decisions in the textiles and apparel goods industry.
**H6:** Pre- and post-COVID-19 and stock market psychology have an insignificant negative impact on investor investment decisions in the consumer electronics goods industry.

### Research method

The current analysis used an event study to analyze market reaction to newly released information from the stock market perspective. “An event study is a statistical technique that estimates the stock price impact of occurrences such as mergers, earnings announcements, and so forth. The basic notion is to extricate the effects of two types of information on stock prices– information that is specific to the firm under question (e.g., dividend announcement) and information that is likely to affect stock prices change in interest rates.” (MacKinlay, 1997). The study sample consisted of companies in the customer products industry that were publicly listed and traded on the Chinese stock markets.

The sample size of the study consisted of market prices at daily closing and stock trading value from 4 months before (−120 days) the outbreak of the pandemic (20 August to 2019 November 2019), the outbreak of the pandemic (20 December 2019), and 4 months after the outbreak of the pandemic (+120 days; January to April 2020) presented in (Figure 3). The daily stock price was used for the accurate calculation to provide more details about the impact of this stock market information. This was a significant methodological shift in event analysis that used regular stock prices rather than monthly stock prices (Kerr et al., 2020). Consumer goods and services businesses were selected because they deliver goods that consumers use regularly.

**Figure 2:**
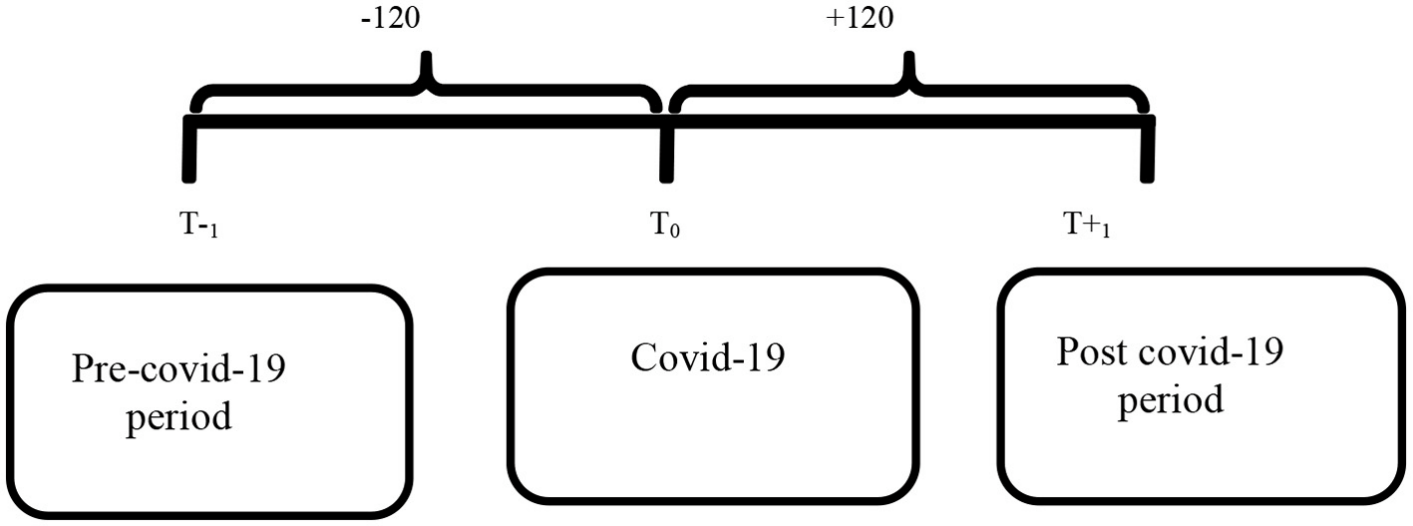
Observation period.

The study used descriptive statistics and multiple regressions and paired *t*-test to compare the two-sample mean differences paired under the assumption that the data were normally distributed. This assessment was used in a repetitive measure that each subject was evaluated twice on the same variable (Badr et al., 2020). The pre and post-COVID-19 analysis had specific characteristics in this type of experimental study. The assessment can be used in a corresponding group configuration in which one or more types of two subjects are paired and are assigned to two different conditions. This design is known as a correlate since the subjects in the groups are paired rather than randomly allocated (Rosenbaum, 2020). We used nonparametric analysis with the Wilcoxon test when the data were not normally distributed. The Wilcoxon signed-rank test is a nonparametric test for detecting substantial variations between two groups of paired data with a specific period. If the normality assumption is not met the Wilcoxon signed-rank test is a paired t-test or t-paired alternative to the paired t-test. The observation period is presented in above Figure 2.

**Figure 3:**
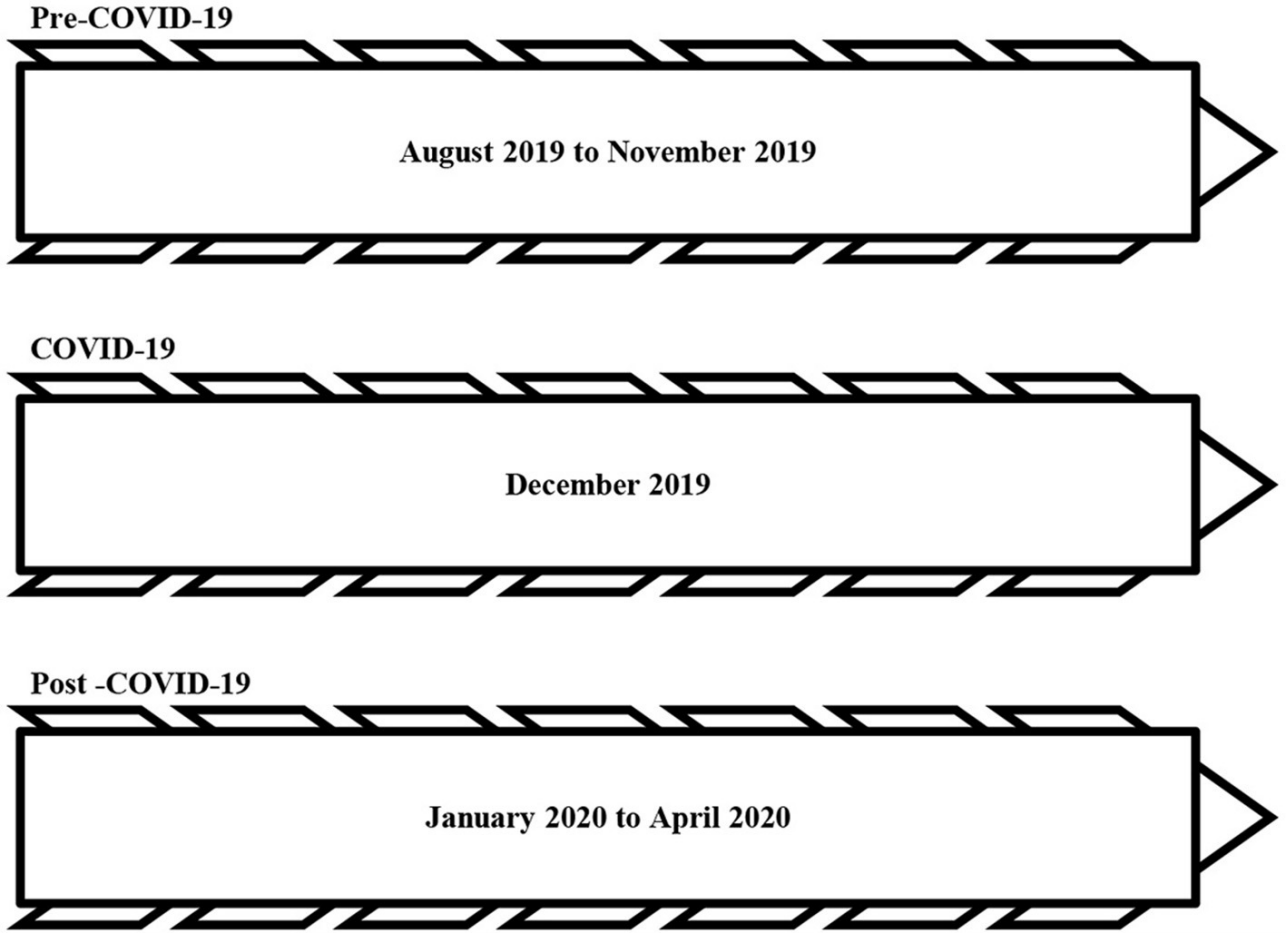
Graphical representations of observation period. Source: Author.

## Result and discussion

The Chinese stock market consists of 21 publicly listed consumer goods industries divided into several subsectors: two companies in the sporting goods industry, one company in the consumer appliances industry, two companies in the cosmetics and beauty products industry, two companies in the household industry, eight companies in the textiles and apparel industry, and two companies in the consumer electronics industry. Each consumer products subsector has its own set of characteristics. The pandemic data were observed 4 months (−120 days) before the outbreak of the pandemic (from August 2019) to 4 months after (+120 days) the outbreak of the pandemic on (20 April 2020). We collected a total of 5,268 observations from 2,634 individuals before the outbreak and 2,634 after the outbreak of the pandemic. The data revealed 210 adverse orders and 2,000 progressive orders for security price (see Wilcoxon signed-rank test, Table 1). The total number of paired observations was 5,268. The sample with another set (post-test) had adverse orders indicating that it was lesser than the first set (pretest). In all, 210 stock prices dropped according to *N* results. Progressive orders denote a sample with another set (post-test) that is greater than the main set (pretest) indicating 46,166 progressive stock price ranges between pre and post-pandemic emerged. This meant that after the outbreak of the pandemic, the stock price declined to 58,039.11. The rate level is represented by the mean rank and the number of rates is represented by the sum of levels. The normal score of negative stock price ranks was 236.75 and positive stock price ranks were 1,418.42.

**Table 1:** Wilcoxon signed ranks test.

		***N***	**Mean rank**	**Sum of ranks**
Post stock	Negative ranks	210^a^	236.75	58,039.11
Price—pre stock	Positive ranks	2,424^b^	1,418.42	3,507,710
Price	Ties	0^c^		
Total		2,634		
Post stock	Negative ranks	261^d^	270.88	80,423.38
Volume—pre stock	Positive ranks	2,373^e^	1,440.10	3,482,608.43
Price	Ties	1^f^		
Total		2,634		

a *Pre security price > post security price*.

b *Pre security price < post security price*.

c *Pre security price = post security price*.

d *Pre security volume > post security volume*.

e *Pre security volume < post security volume*.

f *Pre security volume = post security volume*.

Using the above measure, we found 261 positive and 2,373 negative rank orders, and one tie for the amount of security trading. As a result, the total number of observations for each pair was 2,634. Samples that scored lower in the second group (post-test) than in the first group score received negative ranks (pretest). According to *N* results, the capacity of security trading that experienced a drop totaled 261. Samples that scored higher in the second group (post-test) than in the first group score were given positive ranks (pretest). As a result, the difference between the value of stock transactions and the volume of stock trading pre and post-COVID-19 was 17,675.50, suggesting that the capacity of security trading declined after the outbreak of the pandemic. The ties in the second group score (post-test) were the same as the first (pretest). Negative ranks of the volume of stock trades averaged 270.88% while positive ranks average 1,440.10%.

The *Z* and *P*-value scores were 40.345 and 0.000 as suggested by Wilcoxon signed-rank test (Table 2). Since the value was less than the critical limit of 0.05, this indicates that the stock price before and after the pandemic was significantly different. As a result, there was a positive relationship between the pandemic and stock price.

**Table 2:** Result.

	**Post–pre stock price**	**Post-pre stock volume**
*Z*	−40.345^a^	−39.773^b^
S-Matrix significance (2-tailed)	0.000	0.000

a *Wilcoxon signed ranks test*.

b *Based on negative ranks*.

This result supports the hypothesis that non-economic factors such as the outbreak of a pandemic had a diminutive direct impact on stock price and therefore do not influence investor stock market investment decisions (see Table 3 for research findings by different types of industries).

**Table 3:** Multiple regression of the sporting industry.

**Sporting goods**	***N***	**Rank**	**Ranks**	**Stock**	**Volume**
Post-pre stock prices			
Negative ranks	48^a^	28.17	1,755.48		
Positive ranks	398^b^	249.14	102,209.49		
Relations	0^c^				
Overall	446				
Post-pre stock volume			
Negative ranks	40^d^	38.23	1,946.36		
Positive ranks	406^e^	243.66	102,239.39		
Links	0^f^				
Overall	446				
*Z*				−16.977^b^	−15.909^b^
S-Matrix significance (2-tailed)				0	0
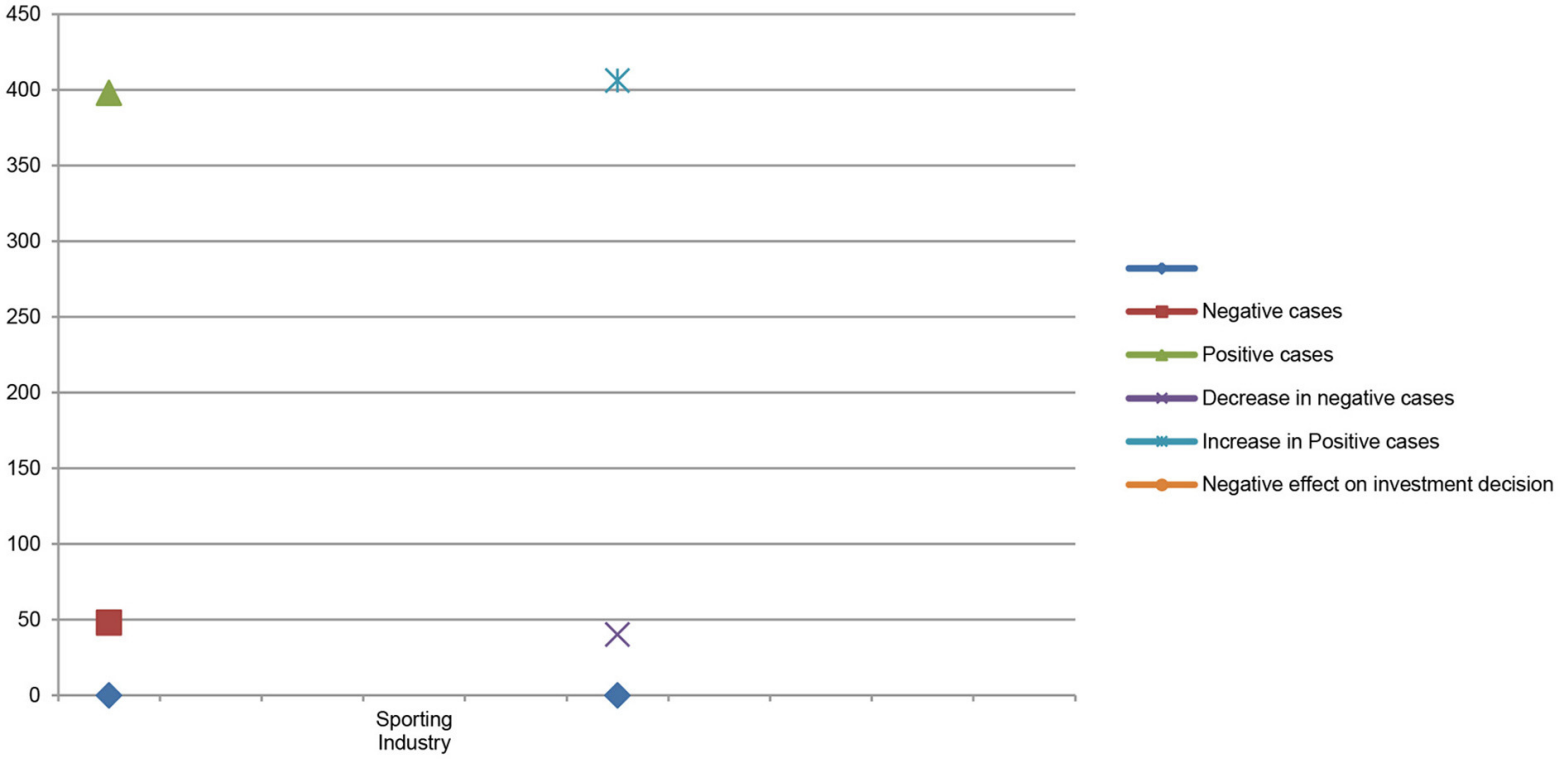					

*The Letter a–f is the alphabets that denote that either security price or volume at pre- pandemic period (Greater, lower or equal) to post- pandemic security price or volume*.

Table 3 shows the comparison between stock market price and stock market trading volume before and after the COVID-19 pandemic and its impact on investor investment decisions in the sporting industry stock market price. The results of multiple regression show that the number of positive cases gradually increased from 398 to 406 in the sporting goods industry due to increases in the number of positive cases. The demands for sporting industry goods and services fell significantly and the production and operation department of the sporting industry went down and most of the business units even shut down their operations as the demand for sporting goods industry declined significantly. During the pandemic period, the sporting goods business indicated sharp variations in stock market price and stock market trading volume before and after the COVID-19 pandemic, and negatively affected investors' investment decisions. This finding is consistent with the findings of Alzyadat and Asfoura (2021).

Table 4 shows the comparison between stock market price and stock market trading volume before and after the COVID-19 pandemic and its impacts on investor investment decisions in the consumer appliances industry stock market price. The results of multiple regression show that the number of positive cases gradually increased from 215 to 217 in the consumer appliances industry due to increases in the number of positive cases the demand for consumer appliance industry goods and services fall significantly. The industry went down even though most of the business units shut down their operations so the demand for consumer appliances declined significantly. During the pandemic period, the consumer appliance industry indicated huge variations in stock market price and stock market trading volume before and after the COVID-19 pandemic which negatively affect investors' investment decisions. This finding is consistent with the findings of Alzyadat and Asfoura (2021).

**Table 4:** Multiple regression of the consumer appliances industry.

**Consumer appliances**		**Rank**	**Ranks**	**Stock**	**Volume**
Post-per stock price			
Negative ranks	4^a^	3.50	57.81		
Positive ranks	215^b^	114.10	26,040.22		
Links	0^c^				
Overall	219				
Post-pre stock volume			
Negative ranks	2^d^	7.30	84.31		
Positive ranks	217^e^	112.92	26,013.72		
Links	0^f^				
Overall	219				
*Z*				−12.128^b^	−12.102^b^
S- Matrix significance (2-tailed)				0	0
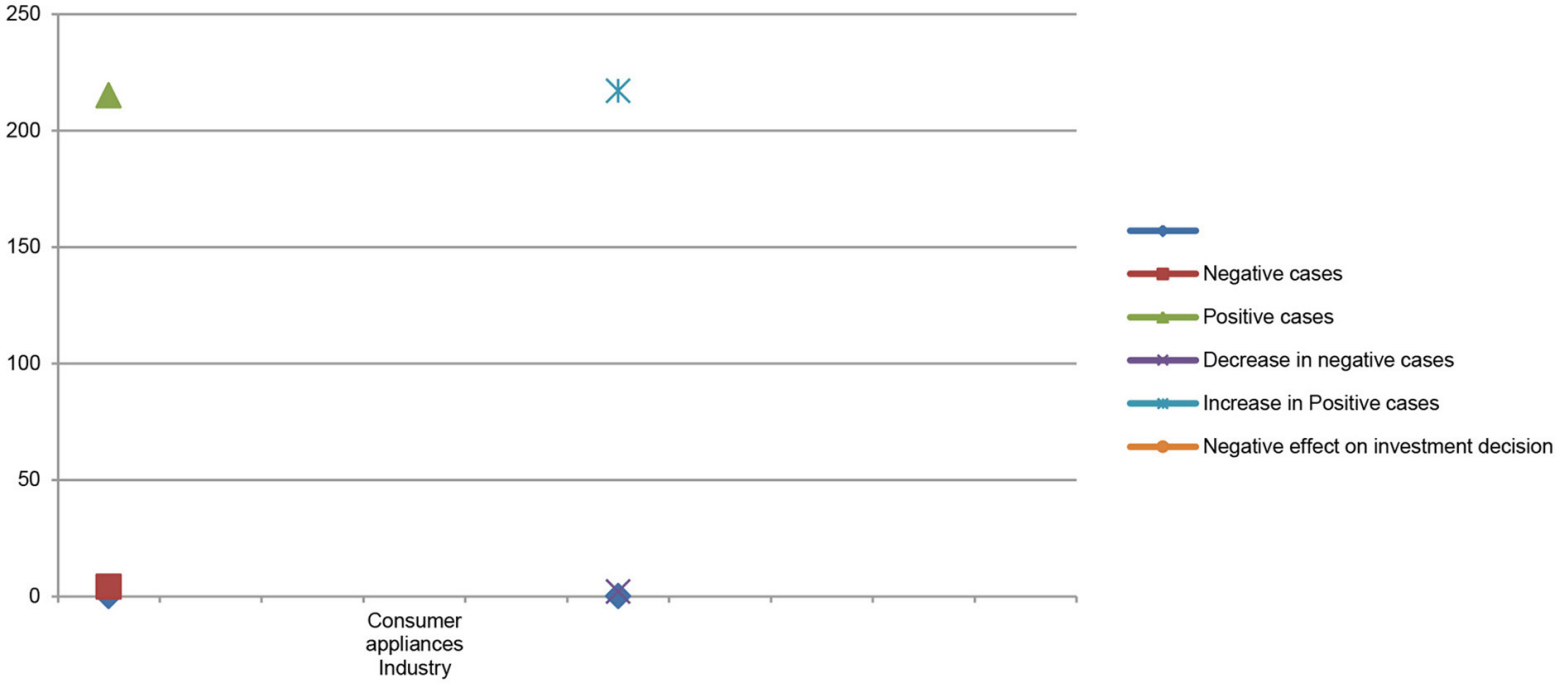					

*The Letter a–f is the alphabets that denote that either security price or volume at pre- pandemic period (Greater, lower or equal) to post- pandemic security price or volume*.

Table 5 shows the comparative difference in stock market price and stock market trading volume before and after the COVID-19 pandemic and its impacts on investor investment decisions in the cosmetic and beauty industry stock market price. The results of multiple regression show that the number of positive cases gradually decreased from 1,170 to 1,139 in the beauty and cosmetics industry. Due to the decrease in the number of positive cases, most beauty and cosmetics businesses show an upward trend in consumer demand for beauty and cosmetics products. Most of the production and operation activities of the beauty and cosmetics industry resumed in a new fashion keeping social, economic, and environmental perspectives in their business overall operations. During the pandemic period, the cosmetic and beauty industry indicated huge variations in their stock market price and stock market trading volume before and after the COVID-19 pandemic, which positively affected investors' investment decisions.

**Table 5:** Multiple regression of the cosmetic and beauty industry.

**Cosmetic and beauty**		**Rank**	**Ranks**	**Stock**	**Volume**
Post-pre stock price			
Negative ranks	122^a^	135.50	18,102.6		
Positive ranks	1,170^b^	701.40	830,340.75		
Links	0^c^				
Overall	1,292				
Post-pre stock volume			
Negative ranks	153^d^	158.50	25,792.72		
Positive ranks	1,139^e^	713.40	821,148.75		
Links	0^f^				
Overall	1,292				
*Z*				−29.038^b^	−28.439^b^
S- Matrix significance (2-tailed)				0	0
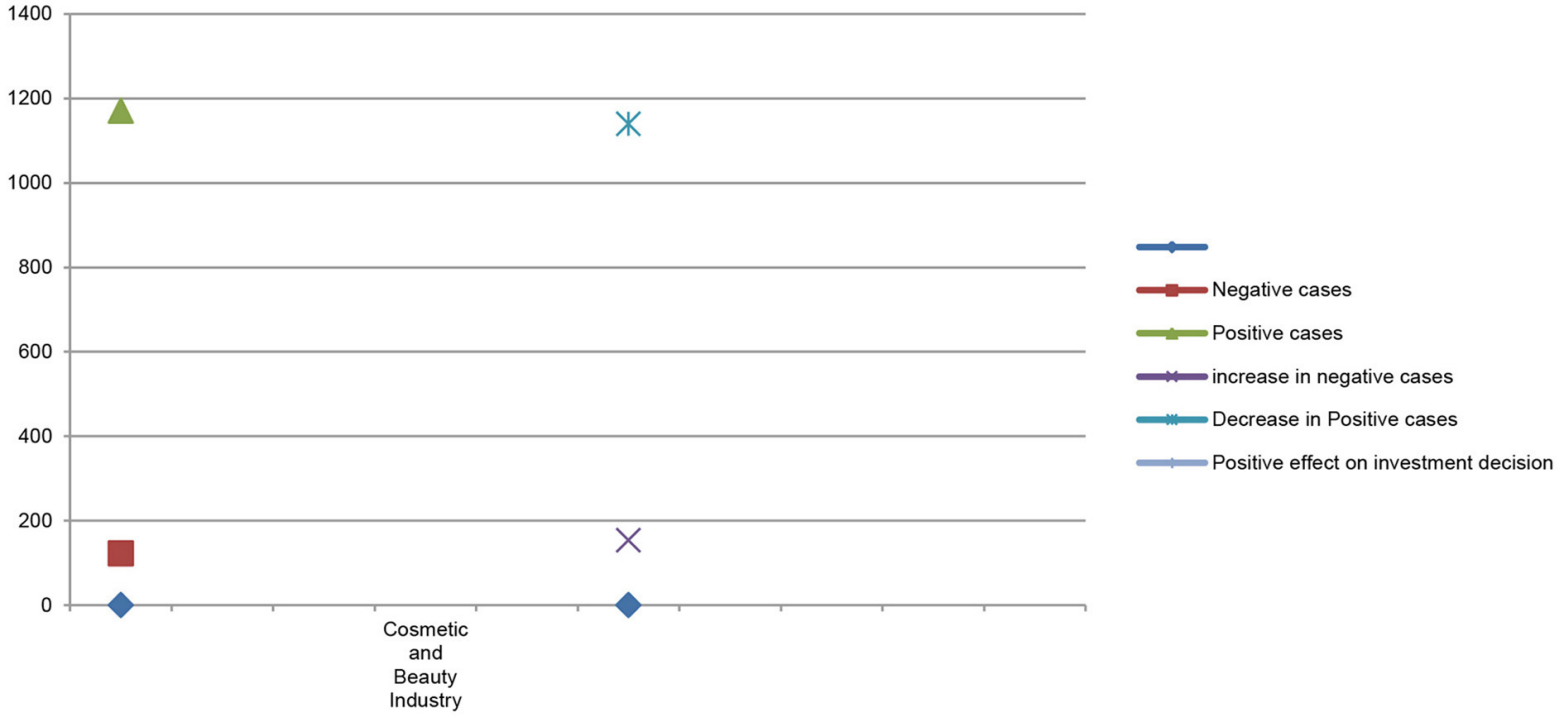					

*The Letter a–f is the alphabets that denote that either security price or volume at pre- pandemic period (Greater, lower or equal) to post- pandemic security price or volume*.

Table 6 shows the difference in stock market price and stock market trading volume before and after the COVID-19 pandemic and its impacts on investor investment decisions in household industry stock market price. The results of multiple regression showed that the number of positive cases gradually decreased from 194 to 182 in the household industry. Due to a decrease in the number of positive cases, the household industry showed an upward trend in consumer demand for household industry products. Most of the production and operation activities of the beauty and cosmetics industry resumed in a new fashion keeping social, economic, and environmental perspectives in their business overall operations. During the pandemic period, the household industry indicated sharp variations in stock market price and stock market trading volume before and after the COVID-19 pandemic, which positively affected investors' investment decisions.

**Table 6:** Multiple regression of household industry.

			**Rank**	**Ranks**	**Stock**	**Volume**
**Household industry**						
	Negative ranks	24^a^	14.5	560.5		
Post-pre stock price	Positive ranks	194^b^	124.40	25,536.5		
	Links	0^c^				
	Overall	218				
	Negative ranks	36^d^	22.5	1,238.6		
Post-pre stock volume	Positive ranks	182^e^	129.40	24,963.9		
	Links	0^f^				
	Overall	218				
*Z*					−11.623^b^	−11.049^b^
S-Matrix significance (2-tailed)					0	0
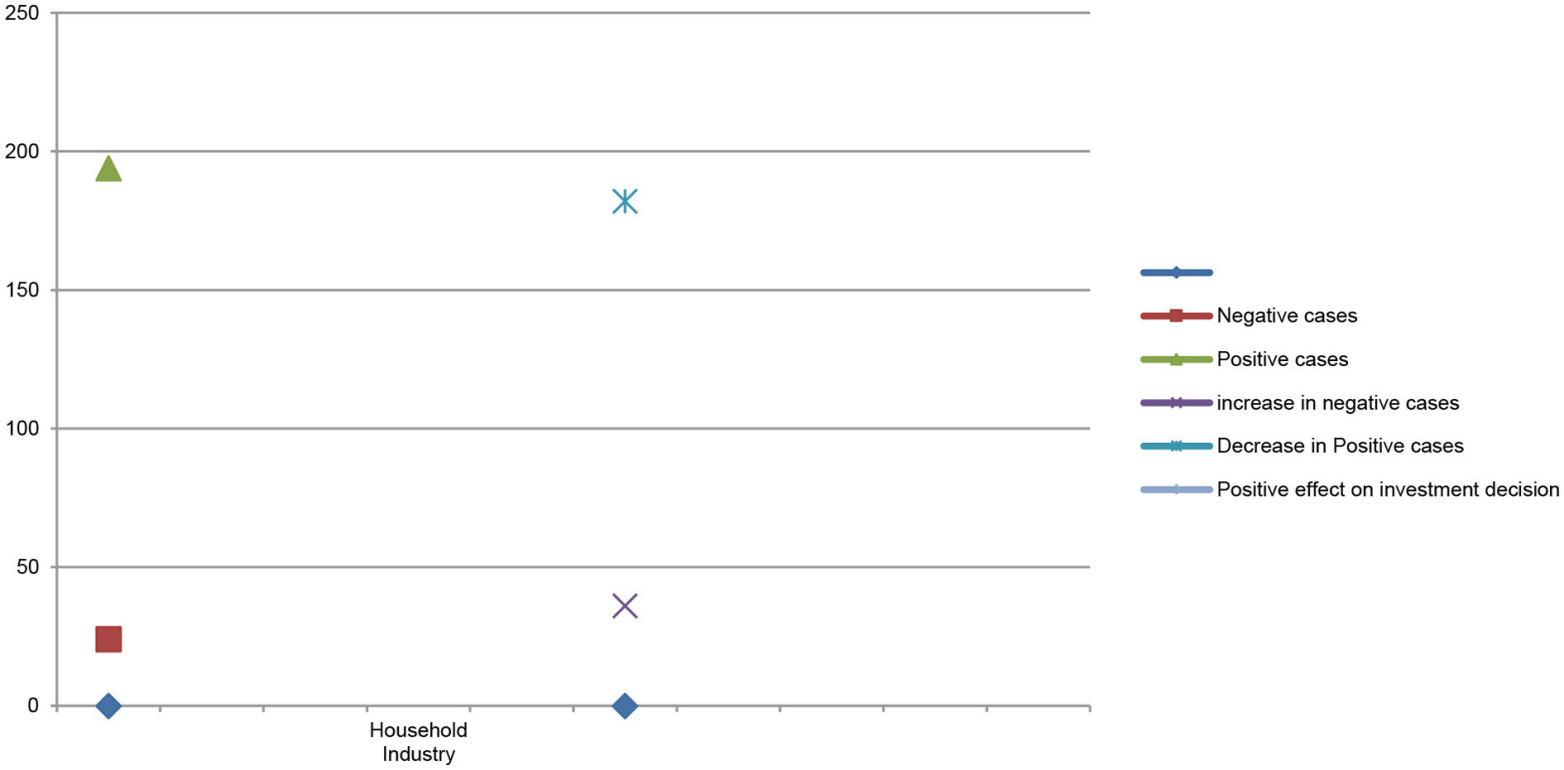						

*The Letter a–f is the alphabets that denote that either security price or volume at pre- pandemic period (Greater, lower or equal) to post- pandemic security price or volume*.

Table 7 shows the difference in stock market price and stock market trading volume before and after the COVID-19 pandemic and its impacts on investor investment decisions in the textile and apparel industry stock market price. The results of multiple regression showed that the number of positive cases gradually decreased from 269 to 253 in the textiles and apparel industry. Due to the decrease in the number of positive cases, most of the production and operation activities of the textiles and apparel industry restarted in a new fashion. Most of the textiles and apparel industries followed social, economic, and environmental considerations in their production and operation activities that gradually captured more consumer demand. During the pandemic period, the textiles and apparel industry indicated huge variations in stock market price and stock market trading volume before and after the COVID-19 pandemic and positively affected investors' investment decisions.

**Table 7:** Multiple regression of textiles and apparel industry.

			**Rank**	**Ranks**	**Stock**	**Volume**
**Textiles and apparel**						
	Negative ranks	7^a^	3.50	83.5		
Post-pre-stock price	Positive ranks	269^b^	144.20	40,666.8		
	Ties	0^c^				
	Total	276				
	Negative ranks	23^d^	14.50	484.5		
Post-pre stock volume	Positive ranks	253^e^	152.20	40,262.8		
	Ties	0^f^				
	Total	276				
*Z*					−13,672^b^	−13,382^b^
S-Matrix significance (2-tailed)					0	0
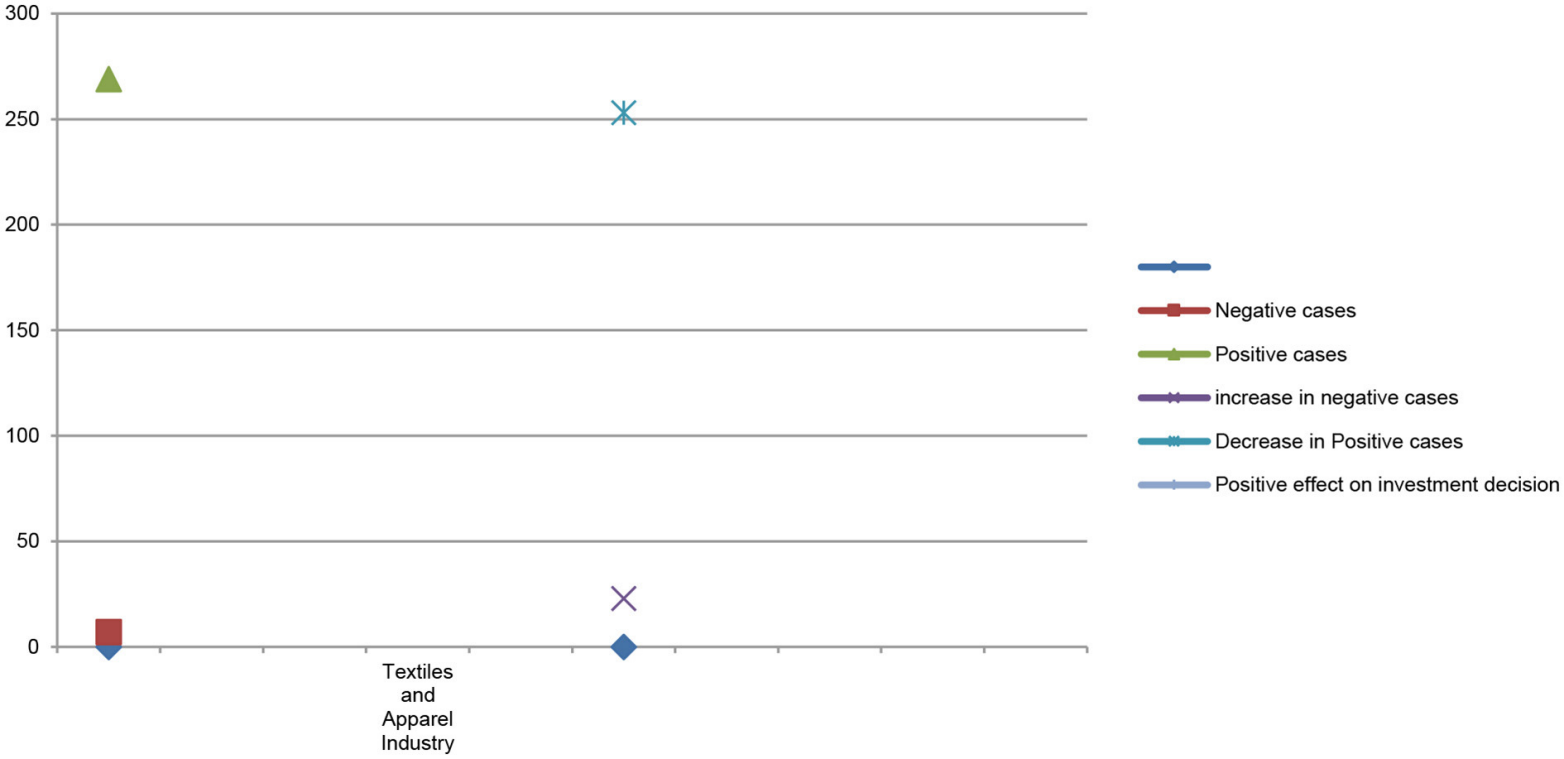						

*The Letter a–f is the alphabets that denote that either security price or volume at pre- pandemic period (Greater, lower or equal) to post- pandemic security price or volume*.

Table 8 shows and compares the difference in stock market price and stock market trading volume before and after the COVID-19 pandemic and its impacts on investor investment decisions in the consumer electronics industry stock market price. The results of multiple regression showed that the number of positive cases gradually decreased from 166 to 161 in the consumer electronics industry. Due to decreases in the number of positive cases, most of the production and operation activities of the consumer electronics industry resumed in a new manner with most of the consumer electronics industries following social, economic, and environmental considerations in their production and operational activities. This gradually captured more consumer demand for the consumer electronics industry. During the pandemic period, the consumer electronics industry showed large variations in stock market price and stock market trading volume before and after the COVID-19 periods, which positively affected investors' investment decisions.

**Table 8:** Multiple regression of the consumer electronics industry.

			**Rank**	**Ranks**	**Stock**	**Volume**
**Consumer electronics**						
	Negative ranks	0^a^	0	0		
Post-pre stock price	Positive ranks	166^b^	81	14,701		
	Links	0^c^				
	Overall	166				
Post-pre stock volume	Negative ranks	5^d^	2	4		
	Positive ranks	161^e^	82	14,698		
	Links	0^f^				
	Overall	166				
*Z*					−10,440^b^	−10,437^b^
S-Matrix significance (2-tailed)					0	0
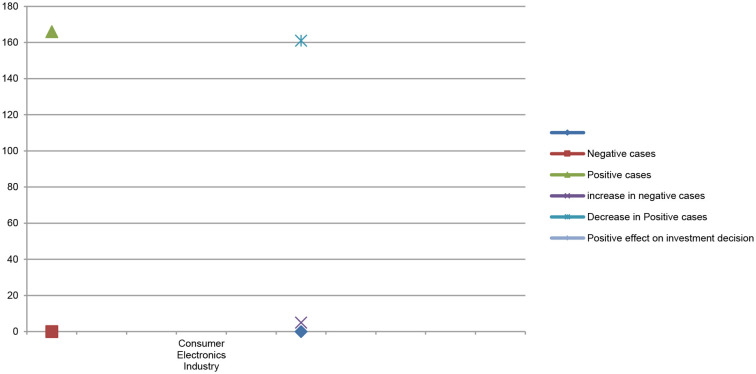						

*The Letter a–f is the alphabets that denote that either security price or volume at pre- pandemic period (Greater, lower or equal) to post- pandemic security price or volume*.

## Discussion

The results of the study indicated that COVID-19 and stock market psychology had a significant positive impact on investor investment decisions in cosmetic and beauty, consumer household, textiles and apparel, and consumer electronics industries. However, only an insignificant negative relationship was found between COVID-19 and stock market psychology and investor investment decisions in the sporting goods and consumer appliance industries. This is consistent with previous studies by Sansa (2020) on the impacts of the pandemic on the stock markets of China and the US. Based on the trend in the number of positive cases, COVID-19 had substantial effects on stock market trends in China. Khan et al. (2020) reported that stock prices dropped by half throughout the pandemic period, but almost improved immediately after the temporary shortage of labor was restored. COVID-19 had a positive and significant correlation with stock price in stock market trading Ramelli and Wagner (2020). However, our results contradicted those of Wang (2015) who reported that the stock markets in China remained strong and stable during the crisis. Although the Chinese stock market continued to be relatively constant in comparison to international markets, our results indicated that the value of A *P* and *Z* scores were 0.000 and 40.345. This supported the research's hypothesis. On the socio-economic observations from this study, the positive environmental effects observed during the pandemic reveal that significant and quick positive change is still possible. This reality should convince citizens and governments all over the world who remain skeptical about environmental and climate change issues that change is vital, urgent, and most important. Further, the results call authorities worldwide to implement new legislation to address global environmental change with macroclimate transformation, environmental degradation, and the imminent possibility of further zoonotic pandemics on earth. The unintended positive effects of the COVID-19 lockdown on global environmental and planetary health are significant even though they will be swiftly undone when the world transitions from lockdowns to a gradual return to normal situations. To have a beneficial impact on the environment, behavioral adjustments such as choosing a lifestyle that decreases carbon footprint are required. Efforts to remediate long-term and continuous environmental pollution remain a mammoth challenge. Simultaneously, observations and knowledge collected of the pandemic's positive environmental benefits should be documented and used to develop comprehensive evidence-based public policies for humanity's survival. World leaders should explore suitable reforms at the national level to sustain the COVID-19 lockdown's good environmental effects. These adjustments would also reduce the chances of another zoonotic disaster.

## Conclusion

The key objective of the study was to find out the difference in stock market price and volume of trading before and after the outbreak of the pandemic and their subsequent impacts on investor investment decisions. Our work contributes to answering a central question: is there any difference in stock market price and volume of the trade before and after the outbreak of COVID-19?

Our results of the study indicate that substantial variations exist in the volume of trading and their subsequent impacts on investor stock market investment decisions before and after the outbreak of the pandemic. Overall, our results revealed that an increase in the positive COVID-19 cases had substantial negative impacts on investors' stock market investment decisions. However, as the number of positive cases decreased, it had a substantial positive impact on investors' stock market investment decisions. According to Machmuddah et al. (2020) the market often responds negatively to short-term events. However, the economy returns to equilibrium and grows steadily in the long run.

To cope with these uncertainties, organizations need to encourage creativity and innovation. Innovation is a continuous mechanism that is generated from within organizations, and it can help shift employee mindsets and prepare them for the future. Long-term innovation strategies will help establish an innovative and creative ecosystem. This would be a very efficient and reliable way of addressing uncertainties and integrating innovative techniques into practice.

Pandemics have a positive correlation with stock prices, and this result is consistent with stakeholder theory. This result is also consistent with Ramelli and Wagner (2020) who stated that COVID-19 had significant positive impacts on stock market prices. However, this result contradicted the findings of Khan et al. (2020) who reported that Chinese stock markets remained unaffected by the COVID-19 pandemic.

The pandemic had a significant impact on industries. As a result, businesses must respond appropriately. Creativity and innovation are required to confront this pandemic. Because of the strategic importance in the evolution of organizations, open innovation has been one of the most highly debated themes in management research for many years. Open innovation will force a company to make the required amendments to secure its long-term survival, and it can ensure an organization's long-term sustainability. Furthermore, open innovation encourages a culture of innovation, learning, and sharing of information. As a result of innovation, organizations would have additional options for responding to challenges such as the pandemic. A learning culture encourages companies to develop their skills while a knowledge-sharing culture increases human resource competence to face these new challenges.

Based on our results, we concluded that the regular closing market price and amount of stock trading were substantially different before and after the outbreak of COVID-19. The stock price and amount of stock trading were influenced by non-financial factors. The results support the efficient market theory which states that a market is more efficient if the information is fully reflected. In practice, the results suggest that while making investment decisions investors should consider these factors. Customer goods companies should be chosen by investors because their goods were in high demand. Further study should be conducted to forecast the pandemic's long-term economic effects.

The study provided several theoretical and practical contributions. Firstly, the study had theoretical contributions to meet the demand of various shareholders. Secondly, the study contributed to the limited literature on the subject by providing new insights on COVID-19 and stock market psychology, fortifying the limitation of traditional financial theories to satisfy the demand of various shareholders in stock markets. Thirdly, the study contributed to the limited literature by identifying the consequences of COVID-19 and stock market psychology and its effects on investor investment decisions in the consumer goods industry. Fourthly the study contributed to identifying individual sectors that were impacted by COVID-19 and related stock market psychology. This was a significant finding given China was the first country to recover from the global pandemic. Finally, the study offers practical implications for different industries in the country.

## Managerial implications

From a practical perspective, this study suggests that investors should be aware of various internal and external factors that impact their investment decisions. The study also helps various organizations, practitioners, and internal and external stakeholders to address the broad impact of domestic and global factors on investor investment decisions in different consumer industries in the pre and post-COVID-19 setting. Similarly, the findings of the study are also helpful for financial institutions that regulate and formulate policies in the post-COVID-19 financial environment. Finally, the findings are also helpful for institutional investors in adopting a broader investment practice that includes a wide array of consumer industries.

## Research limitations and future direction

The generalizability of this study is limited because it was limited to individual consumer industries in the context of China. For further study, a more global sample size could be taken for comparative analysis. Furthermore, it is recommended to conduct a study in other industries such as manufacturing, automobile, e-commerce, and financial services industries. Finally, future research needs to conduct a cross-sectional study to analyze the impact of post-COVID-19 from different industries and countries' perspectives.

## Data availability statement

The original contributions presented in the study are included in the article/supplementary materials, further inquiries can be directed to the corresponding author/s.

## Author contributions

All authors listed have made a substantial, direct, and intellectual contribution to the work and approved it for publication.

## Conflict of interest

The authors declare that the research was conducted in the absence of any commercial or financial relationships that could be construed as a potential conflict of interest.

## Publisher's note

All claims expressed in this article are solely those of the authors and do not necessarily represent those of their affiliated organizations, or those of the publisher, the editors and the reviewers. Any product that may be evaluated in this article, or claim that may be made by its manufacturer, is not guaranteed or endorsed by the publisher.
